# Stress and coping among university faculty and staff at a medical university in the post-pandemic context: a qualitative analysis

**DOI:** 10.3389/fpubh.2025.1674290

**Published:** 2025-12-03

**Authors:** Veselina Slavova, Tsvetelina Tarpomanova

**Affiliations:** Medical College, Medical University “Prof. Dr. Paraskev Stoyanov”, Varna, Bulgaria

**Keywords:** occupational stress, higher education employees, coping strategies, post-COVID-19, faculty well-being

## Abstract

**Background:**

Occupational stress among university faculty and staff has been widely documented, with increasing levels reported globally. The COVID-19 pandemic introduced new pressures, and the resumption of on-site work created reintegration challenges that further affected employees’ well-being. This study aimed to explore the main stressors, coping mechanisms, and strategies to support faculty and staff at the Medical University of Varna (MU-Varna).

**Methods:**

This qualitative study represented the second stage of an investigation into the mental health of MU-Varna employees in the post-COVID period. Data from two focus groups with 24 faculty and staff members were thematically analyzed following Braun and Clarke’s approach. Conventions of trustworthiness (credibility, dependability, confirmability, and transferability) were adhered to in order to ensure rigor.

**Results:**

Four key stress-inducing factors were identified: work overload, problematic workplace relationships, administrative and organizational difficulties, and the need for social contact and support. Participants primarily reported constructive coping strategies, such as seeking social support and problem-solving, although some maladaptive behaviors, including self-blame and self-criticism, were also observed.

**Conclusion:**

This study contributes to the understanding of occupational stress and coping by focusing on post-pandemic reintegration within a medical university context — a setting that combines academic and clinical responsibilities. The findings extend Lazarus and Folkman’s transactional model by revealing culturally and institutionally specific coping responses. The proposed support strategies emphasize training in communication and conflict management, professional development, and team-building to foster resilience and employee well-being in academia.

## 1 Introduction

The study of workplace stress among university faculty and staff has been the focus of numerous investigations worldwide, with a growing body of evidence documenting its increasing prevalence (1–4). Whereas academic work was traditionally associated with autonomy, job security, and consequently high levels of job satisfaction, recent studies have highlighted a reversal of this trend, identifying excessive workload and lack of time (5), as well as a disturbed work–life balance (6), as key contributing factors. Some researchers argue that the perception of excessive workload is shaped by the diverse types of tasks that must be completed within short deadlines. It follows that the multiple and varied roles—teaching, mentoring, research, and administrative responsibilities—assumed by academic staff, combined with high self-expectations, tight deadlines, and insufficient resources, create substantial challenges for the development of a coherent “professional identity” and act as significant drivers of occupational stress (7).

Differences in job roles further shape stress experiences. Academic staff often report high strain due to role conflicts, research expectations, and the perceived incompatibility between teaching and research (8, 9). In contrast, administrative personnel experience stress linked to procedural rigidity, workload intensity, and organizational demands (10). These distinctions underscore the need for context-sensitive approaches to understanding occupational stress in higher education.

The COVID-19 pandemic introduced a new phase, requiring readaptation in the field of higher education. Between 2020 and 2021, a marked increase in stress levels was observed compared to 2019 (11). During the pandemic, additional factors emerged that contributed to heightened stress levels (12). These new challenges were associated with social isolation, the use of novel educational technologies, and the adaptation of curricula and pedagogical models (13). The resumption of on-site work introduced further difficulties, as employees in higher education faced reintegration processes coupled with new governmental and institutional policies imposing increased demands for research productivity and scholarly output. Despite the growing literature, there remains a limited understanding of how faculty and staff in specific regional and institutional contexts have coped with these transitions, particularly in Eastern Europe and medical university settings.

The Medical University of Varna (MU-Varna) provides a particularly relevant case. As a specialized health sciences institution, its employees faced unique pressures, balancing educational, clinical, and research responsibilities. A previous quantitative study at MU-Varna found high levels of emotional exhaustion among approximately half of employees (Maslach Burnout Inventory) and significant correlations between anxiety, depression, and factors such as remote work, perceived stress, and social isolation (Hospital Anxiety and Depression Scale) (14). These findings highlight the need to explore the subjective experiences, stressors, and coping strategies of staff in this context.

To address this gap, a qualitative study using focus groups was conducted to extend insights from the quantitative findings. Qualitative methods are particularly valuable for capturing emotions, perspectives, and interpersonal dynamics, as well as for presenting lived experiences through participants’ statements, reactions, and nonverbal communication (15–17).

According to Lazarus and Folkman’s transactional model (18), coping is a dynamic process through which individuals manage stressful situations, either by addressing the problem itself or regulating emotional responses. This framework provides a useful lens for analyzing how MU-Varna faculty and staff coped with occupational stress in the post-pandemic period.

Building on this foundation, the present study addresses a notable gap in qualitative evidence on post-pandemic reintegration experiences among higher education employees in Eastern Europe. By focusing on the Medical University of Varna, it examines the specific challenges and coping strategies within a medical university context, where academic and clinical responsibilities intersect. Moreover, the study extends Lazarus and Folkman’s model by identifying context-specific coping behaviors influenced by institutional culture and post-pandemic working conditions. These contributions aim to enhance understanding of occupational stress and coping in academia and inform the development of targeted support strategies for university employees.

### 1.1 Objective of the study

The present study aimed to explore the causes of elevated occupational stress, to identify coping mechanisms, and to propose strategies for supporting employees at Medical University of Varna (MU-Varna), Bulgaria.

Two main research questions were formulated:

Research Question 1: What organizational, professional, and psychosocial factors underlie the elevated levels of occupational stress among university faculty and administrative staff in the post-COVID context?

Sub-questions:

What specific organizational factors (e.g., new institutional requirements, lack of resources, administrative procedures) have been identified as stress-inducing in the post-COVID period?
What professional challenges (e.g., role conflicts, incompatibility between teaching and research responsibilities, changes in workload) contribute to elevated levels of stress?
What psychosocial factors (e.g., isolation, anxiety, feelings of insecurity, disturbed work–life balance) are prominent among university employees?
Are there discernible differences in the sources of stress between academic and administrative staff, and if so, what are they?

Research Question 2: What individual and organizational coping strategies are employed by university faculty and administrative staff to manage occupational stress in the post-COVID period, and what additional support mechanisms could be developed to enhance their well-being andprofessional functioning?

Sub-questions:

What individual coping strategies (e.g., personal time management skills, social support, psychological techniques) are employed by university employees?
What organizational support mechanisms (e.g., institutional policies, services, training, team-based support) are perceived as effective by employees?
What difficulties do employees encounter in applying existing coping strategies?
What additional interventions or organizational changes do employees believe would improve stress management and enhance their well-being?

## 2 Materials and methods

### 2.1 Study design

The qualitative study represents the second stage of a broader investigation into the mental health and well-being of university faculty and administrative staff in the post-COVID period, conducted between July and November 2024 at the MU—Varna. The study was designed and implemented in full compliance with the Consolidated Criteria for Reporting Qualitative Research (COREQ) guidelines (19).

### 2.2 Participant selection

The respondents were faculty members and administrative staff at the Medical University of Varna who had previously participated in a quantitative survey conducted between September 2022 and February 2023. An invitation to participate in focus groups was distributed via institutional email to all university employees. A total of 24 participants—17 faculty members and 7 administrative staff from various faculties—responded to the invitation. These participants were organized into two focus groups of 12 participants each, with each group including a mix of academic and administrative staff to encourage diverse perspectives and interaction. Each focus group participated in two separate sessions to allow for in-depth discussion and exploration of the topics. Participation was entirely voluntary and was confirmed by signing an informed consent form. The organization of the group discussions adhered to established standards for conducting this type of qualitative inquiry (20).

### 2.3 Data collection

The group discussions were conducted in the form of semi-structured interviews with open-ended questions, held on the premises of the Medical University of Varna at the end of the participants’ working day. The two researchers alternated in moderating the discussions and taking notes. Each session lasted between one and one and a half hours.

The role of the moderator was to guide the discussion, encourage participants to express their opinions, and pose clarifying questions to facilitate a more in-depth analysis of the collected information.

At the request of some participants, no audio or video recordings of the sessions were made. Instead, detailed field notes were taken during the focus group discussions. To ensure accuracy, the notes were reviewed and supplemented by both researchers after each session.

At the beginning of each session, the rules of group interaction were explained. Participants were informed about the purpose of the study and the potential benefits of the research. Confidentiality of the discussions was guaranteed, with each participant assigned an identification code — faculty members were designated with the letter P and administrative staff with the letter S. Before the start of the discussions, all participants signed an informed consent declaration.

Data were analyzed using thematic analysis, following the six-phase framework proposed by Braun and Clarke (21), which includes familiarization with the data, generation of initial codes, searching for themes, reviewing themes, defining and naming themes, and producing the report. Participants’ responses were coded and analyzed using NVivo-15 software to identify recurring patterns, from which the main themes were derived.

To ensure the trustworthiness of the study, we followed established criteria for qualitative research. Credibility was supported through careful thematic analysis, iterative discussions within the research team, and validation of emerging themes against participants’ accounts. Transferability was facilitated by providing rich descriptions of the institutional context and participants’ perspectives. Dependability was strengthened by maintaining detailed documentation of data collection and analysis procedures, while confirmability was enhanced through systematic coding and collaborative interpretation, minimizing individual researcher bias. In addition, the research team engaged in reflexivity throughout the study, acknowledging their dual role as members of the academic community and researchers. Regular team reflections and critical discussions were undertaken to enhance awareness of potential assumptions and to ensure that the voices of participants remained central to the analysis.

The study was conducted in accordance with the principles of the Declaration of Helsinki and was approved by the Research Ethics Committee of the Medical University of Varna (Protocol No. 119/21.07.2022).

## 3 Results

### 3.1 Participant characteristics in the focus groups

The study included 24 employees of the Medical University of Varna, of whom 17 were faculty members and 7 were administrative staff. The composition of the two groups was determined either by participants’ preference or randomly. Two groups were formed, each comprising 12 participants—both faculty and administrative staff. Each group participated in two sessions, for a total of four sessions.

The first group consisted of employees from the same university department, who already had established interpersonal relationships. The second group had a mixed composition—some participants were colleagues who knew each other, while others were not previously acquainted. Both groups included participants who occupied different hierarchical positions.

### 3.2 Main themes

Using NVivo-15 software, the frequency of words used in the discussions was analyzed. While describing their feelings and experiences, participants most often mentioned the word *“work,”* followed by *“stress,” “tasks,” “overload,”* among others. This finding informed and enriched the thematic analysis (see Table 1).

**Table 1:** Word frequency.

Word/phrase	Frequency	Percentage
Work	22	20.0%
Stress/stressful	11	10.0%
Tasks	10	9.1%
Overload	9	8.2%
Teaching/teach	8	7.3%
Infected/infection	7	6.4%
Projects	7	6.4%
Problem(s)	6	5.5%
Time	6	5.5%
Support/supported	4	3.6%
Tension/tense	4	3.6%
Burnout	3	2.7%

Following a careful reading of the field notes by both authors, thematic analysis conducted with NVivo-15 grouped together recurring or semantically similar statements such as *“a lot of diverse tasks were dumped on us,” “I felt overwhelmed,” “I had too much work,”* which were consolidated under the theme *“work overload.”*

In this way, for each of the two research questions, codes were created based on direct excerpts from participants’ statements, which were subsequently synthesized and ranked in importance according to the frequency of mention. Each participant gave more than one answer. The responses were coded into different groups, according to the meaning of the individual parts of the statements (Table 2).

**Table 2:** Factors of occupational stress.

Factor of occupational stress	Responses (n)	Percentage (%)
Work overload	12	50%
Workplace relationship problems	12	50%
Administrative and organizational challenges	11	46%
Need for social contacts and support	7	29%

### 3.3 Work overload

Half of the respondents (12 of 24) reported experiencing work overload during and after the COVID-19 pandemic. The difficulties associated with distance teaching—which required more preparation time and specific technical skills—were perceived by some faculty members as a serious challenge contributing to an increased sense of workload. Participants noted that they exerted more effort and needed more time to prepare their lectures and organize examinations. Even though the institution supported their work through continuous training, some felt they spent disproportionately more time on tasks they used to complete faster:

*“There was always something to do—if I wasn’t in training, I was teaching, or on the phone, or updating my lectures or preparing exams. I felt like 24 h in a day were not enough.”* (P15, Age 48, Associate Professor, Group 2).

After the adaptation period, the sense of overload persisted as regular teaching duties were combined with resumed research, publication tasks, and administrative responsibilities. For example, one lecturer shared:

*“I’m a course coordinator, I’m preparing the accreditation documents for the department, I participate in projects, and have to write articles too.”* (P9, Age 52, Associate Professor, Group 2).

This highlights role conflict and role accumulation — simultaneous academic, administrative, and project commitments that blurred professional boundaries. Such fragmented work responsibilities made it difficult for employees to sustain a coherent professional identity, which in turn reinforced their perception of overload.

Administrative staff also described increased pressure, though from a different angle. An administrative employee noted that *“faculty members have very high demands regarding cleanliness… the work is the same as before, but now they expect more.”* (S3, Age 51, Laboratory Technician, Group 1).

In several narratives, participants reported a persistent spillover of work responsibilities into personal life, resulting in work–life imbalance and emotional guilt.

*“I usually work on my dissertation and publications in the evenings at home, which makes me feel guilty toward my family.”* (P13, Age 32, Assistant Lecturer, Group 2).

Overall, the data indicate that post-pandemic workload at the Medical University of Varna extended beyond teaching duties and reflected both increased institutional expectations and individual striving for professional adequacy.

### 3.4 Workplace relationship problems

Half of the respondents (12 out of 24) associated workplace stress with conflicts in relationships with colleagues and students. This issue was particularly evident in the context of organizations with heterogeneous hierarchical structures. Several causes were identified by participants, including competition among colleagues, differences in experience and tenure, varied interests, and the influence of power positions.

Competitive relationships and conflicts arising from differing views and experience were also highlighted as sources of tension and stress. A young faculty member shared that they tried to propose a more efficient way to accomplish a task but were met with rejection and even negative feedback from a senior colleague.

*“Not only do I fulfill my duties, but I try to ease the workload of other colleagues. I suggested a faster method to a colleague, but she firmly rejected it, insisting we stick to the old way. Perhaps because she has more experience, she seemed offended and stopped communicating with me.”* (P13, Age 32, Assistant Lecturer, Group 2).

Conversely, senior staff described tension arising from perceived imbalances in work ethics and task distribution:

*“Some colleagues neglect additional responsibilities related to project activities, which are a priority for the university. They focus only on their immediate tasks and stop there.”* (P4, Age 44, Senior Lecturer, Group 1).

Administrative staff also reported relationship strain, though their concerns centered on task ambiguity and overlapping responsibilities rather than hierarchy.

*“It can be frustrating when tasks are duplicated or responsibilities are not clearly defined.”* (S3, Age 51, Laboratory Technician, Group 1).

Unlike faculty members, who focused on recognition and fairness, administrative employees primarily experienced role ambiguity as the main stressor. This reflects differences in coping orientation: while academic personnel often attempt to assert control or seek validation, administrative staff typically rely on problem-focused strategies, such as clarifying duties or improving coordination.

### 3.5 Administrative and organizational challenges

Eleven out of 24 participants mentioned problems of an administrative and organizational nature, related to uneven workload distribution and limited time to complete tasks.

This theme was interpreted differently by faculty and staff. Faculty members, as previously noted, juggle multiple roles. They deliver lectures and exercises, and conduct assessments, carry administrative duties, serve as course coordinators, prepare schedules, monitor the educational process, and respond to issues during the semester. Some are also administrative assistants responsible for entering planned and actual teaching loads of all lecturers in electronic platforms for their department or faculty sector. Concurrently, they must prepare scientific publications, participate in projects, and various initiatives during the academic year. Assistant professors are also required to prepare and defend a dissertation within three years. From an organizational perspective, it was suggested that a more equitable distribution of tasks among all staff could reduce work-related stress.

*“I fully understand colleagues who feel overloaded because I work at the hospital, am an administrative assistant, and also head the academic sector. I think these functions are too many for one person. I really want someone else to take over part of my work — for example, to be an administrative assistant.”* (P11, Age 56, Associate Professor, Group 2).

Those fulfilling administrative assistant roles reported difficulties caused by delayed deadlines for submitting information and late access to the online platform for entering teaching loads, which hindered planning summer vacations.

*“I am an administrative assistant, for example, and I am responsible for entering all colleagues’ hours into the system, but I still have not been granted access by the administrator. My permission to take leave depends on this.”* (P12, Age 54, Associate Professor, Group 2).

Clarifying role boundaries and optimizing administrative processes could therefore play a key role in reducing occupational strain and enhancing job satisfaction among faculty members.

### 3.6 Need for social contacts and support

Almost one-third (7 out of 24) of participants identified social isolation and lack of contact as serious challenges during the COVID-19 pandemic, in addition to remote work. While the transition to remote work enabled academic continuity, it simultaneously disrupted the sense of belonging and collegial connectedness that characterizes university life. Some faculty described their work as “difficult” and “lonely” due to the lack of visual contact with students and personal interaction with colleagues. They felt relieved upon returning to the workplace.

*“I am used to sharing with my colleagues, and during that period, we only spoke on the phone, which is not the same. When I returned to the university, I felt relieved — I see the students face to face and talk to many more colleagues.”* (P1, Age 49, Associate Professor, Group 1).

This quote captures the psychological importance of face-to-face interaction as a source of emotional energy and motivation.

Administrative staff, even those not fully remote, similarly highlighted the restorative function of in-person contact. One participant noted feeling “more empathy and support” when surrounded by colleagues (S2, Age 53, Administrative Officer, Group 1).

During the discussion, respondents emphasized that colleague support was crucial in stressful situations caused by workload accumulation, conflicts, and organizational problems. For some, emotional support — understanding and empathy — helped release emotions and reduce tension. For others, support was manifested as concrete help in completing tasks.

*“I have a close colleague and often spoke to her on the phone to share how I felt. When I returned, daily work distracted me from thoughts about my illness diagnosis, and I felt calmer. Besides, I felt supported by my colleagues.”* (S2, Age 53, Administrative Officer, Group 1).

Beyond the pandemic context, many respondents emphasized that collegial support remained a key coping mechanism in managing everyday work stressors such as workload accumulation and organizational challenges. Emotional support—expressed through understanding and sharing experiences—served as a buffer against tension, while instrumental support, such as direct assistance in completing tasks, fostered a sense of solidarity.

### 3.7 Coping strategies for workplace stress

Participants described a range of coping strategies reflecting both problem-focused and emotion-focused approaches (18). After thematic coding, several dominant behavioral patterns emerged, illustrating how university employees respond to professional stressors in diverse but often complementary ways.

### 3.8 Seeking support from colleagues

The most frequently mentioned coping mechanism (9 of 24 participants) involved seeking advice, assistance, or emotional support from colleagues. For many, collegial relationships functioned as both practical and emotional resources. Faculty members often turned to more experienced colleagues for help with specific issues, while others valued empathy and understanding in stressful moments.

As one participant explained: *“When something is unclear to me, I go and ask someone who gives me advice on how to handle the situation.”* (P8, Age 42, Senior Lecturer, Group 2). This behavior aligns with social support theory, suggesting that perceived availability of help and empathy can buffer the effects of workplace stress and foster a sense of belonging.

### 3.9 Self-blame and self-criticism

Six participants reported self-blame as a recurring emotional response to workplace difficulties. Rather than externalizing stress, they tended to internalize it, questioning their competence or feeling guilty for work-life imbalance.

One participant shared: *“I usually blame myself, and when I’m reproached for not doing something or doing it incorrectly, I take it at face value.”* (P15, Age 48, Associate Professor, Group 2).

This pattern illustrates an emotion-focused coping strategy that, while promoting self-reflection, may also heighten vulnerability to stress-related exhaustion and psychosomatic symptoms.

### 3.10 Planning and problem-solving

A smaller but distinct group (5 participants) emphasized structured planning, prioritization, and task management as ways to control workload and reduce anxiety.

As one administrative officer described: *“When I have many duties, I prioritize them and plan to complete them in that order.”* (S4, Age 46, Group 1).

These responses reflect problem-focused coping, aimed at directly modifying the stressor through organization and time management. Participants who used such strategies often reported a greater sense of control and effectiveness.

### 3.11 Individual self-help and rationalization

Four respondents relied on individual self-help methods—reading specialized literature, introspection, or cognitive reframing—to manage emotional tension. This group demonstrated a preference for autonomous, cognitive coping, consistent with self-regulation theory.

For example, one participant shared: *“I bought several self-help books and discovered interesting things about myself.”* (P2, Age 43, Associate Professor, Group 1).

Such strategies emphasize personal growth and meaning-making, transforming stress into an opportunity for self-understanding.

### 3.12 Avoidance and postponement

A few participants (3 of 24) described avoidance or delay as coping mechanisms. They tended to postpone dealing with stressors or avoided potential conflicts until “the right moment.”

As one participant noted: *“I do not rush to do something. I’ve noticed that when I wait for the right moment, things fall into place.”* (P10, Age 38, Senior Lecturer, Group 2).

While avoidance may temporarily reduce emotional discomfort, it often prevents effective problem-solving and can prolong tension over time.

The identified coping strategies reveal a dynamic interplay between interpersonal and intrapersonal resources. Socially oriented strategies—such as seeking support—dominate, suggesting that collegial networks at the Medical University of Varna play a central role in emotional regulation and resilience. At the same time, self-critical and avoidance tendencies highlight individual vulnerabilities and the potential emotional costs of professional dedication.

These findings confirm that balanced coping repertoires, combining problem-solving and emotional regulation, are essential for sustaining well-being in academic settings. The insights also underline the importance of institutional support systems (e.g., peer mentoring, stress management workshops) that foster adaptive coping and reduce isolation.

### 3.13 Institutional support for reducing professional stress

After sharing their usual coping methods, participants discussed possibilities for institutional support to reduce work stress. Members of the first group reported that before the COVID-19 pandemic, their faculty organized team-building activities such as excursions, themed gatherings, cooking contests, etc. Four people supported these practices and hope they will be restored.

Seven faculty members and three staff noted the need for training and group discussions on common causes of workplace conflicts. They discussed potential specialized trainings on communication skills, conflict management, constructive stress coping strategies, and more. These activities are expected to be organized by the university at convenient times for employees.

Two faculty members emphasized the need for additional professional training, also to be offered and funded by the university. This would both help reduce workload and prevent burnout, as well as improve professional development, which benefits the institution.

## 4 Discussion

This study explored the main sources of workplace stress and coping strategies among university faculty and staff in the post-pandemic context at the Medical University of Varna. The findings reaffirmed several stressors identified in the international literature—such as work overload, administrative burden, and interpersonal conflict—while also revealing contextual and cultural specificities that contribute new insights into how these processes unfold in Eastern European academic settings (Table 3).

**Table 3:** Ways of Coping with Workplace Stress.

Way of coping	Responses (*n*)	Percentage (%)
Seeking help from colleagues	9	37.5%
Self-blame and self-criticism	6	25%
Planning and problem-solving	5	20.8%
Self-help	4	16.7%
Procrastination/avoidance	3	12.5%

The results can be interpreted through the lens of Lazarus and Folkman’s (18) transactional model of stress and coping, which conceptualizes stress as a dynamic process of appraisal and response. Participants’ experiences illustrate both primary appraisal (perceiving workload, conflict, and organizational issues as threats to well-being) and secondary appraisal (evaluating available coping resources). Faculty members, for instance, often perceived excessive demands and unclear role expectations as exceeding their capacity—triggering emotional reactions such as guilt and self-doubt. The reliance on social support, planning, and time management reflects problem-focused coping, whereas self-blame and avoidance behaviors align with emotion-focused coping.

Theoretical models of occupational stress posit that stress arises when employees face high demands alongside insufficient coping resources (7). While previous research has consistently identified workload, role conflicts, and lack of support as major stressors, much of it has focused on either pre- or post-pandemic snapshots. The present study adds to the literature by capturing lived experiences during the reintegration phase following COVID-19, offering qualitative evidence from an underrepresented context—an Eastern European medical university—where cultural and organizational features shape stress and coping in distinct ways.

### 4.1 Work overload and organizational demands

Consistent with prior research, work overload emerged as the most prevalent stressor (22–25). Participants reported that excessive teaching, research, and administrative obligations led to emotional strain and fatigue. Previous studies have demonstrated that juggling multiple academic roles contributes to heightened stress and exhaustion (26, 27), while research in India similarly identified administrative and research-related duties as major stressors due to role incompatibility and ambiguity (28, 29).

The current findings extend existing evidence by showing that even after returning to in-person work, the sense of overload remained. This persistence suggests that post-pandemic institutional adjustments failed to mitigate long-standing structural pressures. The data further reveal that blurred role boundaries and limited institutional flexibility intensified workload stress—reflecting specific features of the Bulgarian higher education system. These insights add a contextual and cultural dimension to global discussions of academic stress.

### 4.2 Workplace relationships and hierarchical dynamics

Conflicts in workplace relationships were another major source of tension. As in other studies, competition, status differences, and power imbalances were frequently cited as triggers for stress (30–32). The literature emphasizes that poor communication and lack of trust undermine cooperation and collective performance (33, 34).

However, this study highlights that hierarchical organizational culture and generational gaps within Bulgarian academia exacerbate such conflicts. Participants often described difficulties in communication between senior and junior faculty, shaped by traditional respect for authority and status. Unlike findings from more egalitarian academic systems, these dynamics reflect a culturally embedded hierarchy that influences not only interpersonal relations but also stress appraisal and coping choices. This provides a novel contribution by contextualizing workplace stress within local cultural norms and institutional structures.

### 4.3 Social support and collegiality

Social contact and collegial support emerged as critical resources for managing workplace stress, consistent with prior research linking perceived support to improved psychological well-being (35–41). Respondents described relying on instrumental, informational, and emotional support from colleagues.

What distinguishes the present findings is the centrality of collegial relationships as both a coping strategy and a resilience mechanism. Within the collectivist and interdependent professional culture of the Medical University of Varna, peer support appears to compensate for limited formal institutional structures addressing well-being. Thus, the study underscores the relational dimension of coping, expanding current models that often prioritize individual-level strategies.

### 4.4 Coping strategies and professional development

Following Lazarus and Folkman’s (18) typology, participants reported using diverse coping approaches: Problem-focused coping (s eeking help, planning, self-help), Emotion-focused coping (social support, emotional sharing), and Avoidance-based coping (self-blame, procrastination).

Constructive coping predominated, though participants acknowledged limited access to formal training in stress management. Prior studies emphasize the role of institutional training and professional development as protective factors against burnout (22, 40, 42). Indeed, inadequate training has been shown to reduce engagement and heighten workload-related stress (43–45). In contrast, our results suggest that insufficient social support and problematic workplace relationships were more significant stressors for participants. While professional training remains important, these findings highlight the protective function of collegial and cultural support, emphasizing that well-being initiatives must integrate both personal and relational dimensions.

### 4.5 Contribution and implications

This study contributes to the growing literature on occupational stress by situating well-documented stressors within the organizational and cultural context of Eastern European academia. It demonstrates that in such environments, hierarchical structures, multiple role expectations, and strong interpersonal interdependence create unique stress patterns but also enable relational coping mechanisms that buffer psychological strain.

The findings suggest that effective institutional interventions should go beyond workload reduction to include: structured mentoring and peer-support systems; targeted training in communication, emotional intelligence, and conflict management; and participatory management practices that reduce hierarchical barriers.

By integrating culturally sensitive approaches with organizational reforms, universities can enhance employee well-being and resilience, contributing to both individual and institutional sustainability.

### 4.6 Limitations of the study

This study contributes to expanding knowledge on mental health and workplace well-being. It was conducted at a single public university, so the results cannot be generalized to all higher education institutions in Bulgaria. Universities with different funding models and profiles were not examined, which could be the subject of future research.

The focus groups included faculty and staff from various faculties and administrative units of the university, but not from all. Some units showed greater participation than others. Faculty members predominated over administrative staff, which may have influenced the identification and ranking of the main themes.

It is also possible that some participants did not fully express their opinions during the group discussions. Future studies should consider combining focus groups with individual interviews.

## 5 Conclusion

This study identified the key sources of workplace stress in the post-COVID period at the Medical University of Varna and the coping strategies most commonly used by faculty and administrative staff. Through Braun and Clarke’s thematic analysis, four central stress factors were delineated: work overload, problematic workplace relationships, administrative and organizational difficulties, and the need for social contact and collegial support. These findings reaffirm globally recognized stressors while also highlighting contextual features specific to medical higher education and the Eastern European academic environment.

Interpreted through Lazarus and Folkman’s transactional model, the results demonstrate how employees appraise stressors and mobilize coping resources, with most participants employing constructive, problem-focused strategies alongside emotional and avoidance-based responses. The combination of institutional challenges and strong reliance on peer support underscores the importance of organizational culture in shaping coping behavior.

By identifying both stressogenic factors and coping resources, this study contributes new insights into the reintegration of university employees after the pandemic—a topic insufficiently explored in Eastern European medical institutions. The findings support the development of targeted policies for employee well-being. Recommended institutional measures include specialized training in communication and conflict management, enhanced professional development opportunities, and initiatives that strengthen social connectedness and teamwork. Such interventions may help mitigate occupational stress and reinforce a supportive and resilient academic environment.

## Data Availability

The original contributions presented in the study are included in the article/supplementary material, further inquiries can be directed to the corresponding author.
